# Integrated CIRCLE-seq with RNA-seq to decipher the quantity, localization, and functional features of eccDNA in AML

**DOI:** 10.3389/fonc.2025.1701989

**Published:** 2025-11-07

**Authors:** Lijuan Gao, Qiongyu Lu, Hao Li, Changgen Ruan, Zhao Zeng, Suning Chen

**Affiliations:** 1 National Clinical Research Center for Hematologic Diseases, Jiangsu Institute of Hematology, The First Affiliated Hospital of Soochow University, Suzhou, China; 2 Institute of Blood and Marrow Transplantation, Collaborative Innovation Center of Hematology, Soochow University, Suzhou, China; 3 Cyrus Tang Hematology Center, Soochow University, Suzhou, China; 4 Department of Clinical Laboratory, No. 989 Hospital of Joint Logistic Support Force of The Chinese People's Liberation Army (PLA), Pingdingshan, China

**Keywords:** circle-seq, AML, RNA-seq, eccDNA, prognosis

## Abstract

**Background:**

Extrachromosomal circular DNA (eccDNA) plays critical roles in cancer, yet its landscape in acute myeloid leukemia (AML) remains unexplored.

**Methods:**

We used CIRCLE-seq and RNA-seq to characterize eccDNA in 12 AML patients and 4 healthy controls.

**Results:**

AML cells showed significantly increased eccDNA counts and gene involvement versus healthy controls, with distinct size peaks at 202 and 368 bp. eccDNAs localized non-randomly to chromosomes 1, 2, and 10–19, enriching near transcription start sites and regulatory regions. Functional analysis revealed activation of oncogenic pathways (e.g., MAPK, ErbB signaling) in AML-associated eccDNA. Integrative analysis identified 570 genes upregulated at both the eccDNA and mRNA levels, including myeloid leukemia-related genes (e.g., *FLT3*, *RUNX1*, and *CD33*) and oncogenes. Prognostic analysis showed that high expression of these genes correlated with poor outcomes in AML.

**Conclusions:**

This study unveils the eccDNA landscape in AML by direct CIRCLE-seq, linking its accumulation to transcriptional dysregulation and leukemogenesis, and highlights eccDNA as a potential biomarker and therapeutic target.

## Introduction

Extrachromosomal circular DNA (eccDNA) has emerged as a critical frontier in cancer biology, primarily due to its diverse roles in oncogene amplification, transcription regulation, tumor heterogeneity, and drug resistance and its potential as both a prognostic marker and a therapeutic target (1–3). These unique nucleic acid molecules exhibit a broad size spectrum, ranging from dozens of base pairs (bp) to kilobase- and megabase-pair scales. Based on their size and characteristics, they are classified into several subtypes, including small polydispersed DNA (spcDNA), microDNA, telomeric circles, extrachromosomal rDNA circles (ERC), and ecDNA, and they are widely distributed in human normal tissues, cancerous tissues, and body fluids (3–6). Their biogenesis is intricately linked to genomic instability events, including chromosomal rearrangements/fragmentation (e.g., chromothripsis), DNA repair (e.g., homologous recombination and non-homologous end joining), and replication stress, although the exact mechanisms remain unclear (3, 7, 8). Functionally, eccDNAs could provide effective means for gene amplification by increasing the copy number directly, acting as trans-acting factors like super-enhancers, or enhancing chromatin accessibility via their circular structure (3, 9).

In hematological malignancies, however, our understanding of eccDNA lags significantly behind that of solid tumors, despite its potential relevance to leukemogenesis. A few studies showed that eccDNAs are common in normal hematopoietic and acute myeloid leukemia (AML) cells (10), although a previous study indicated that eccDNA amplification did not occur in blood or normal tissue (2). Consistently, cytogenetic studies have shown that double minutes (DMs, a kind of eccDNA) are rare in myeloid neoplasms but associated with micronuclei, *MYC* and *KMT2A* amplification, complex and monosomal karyotypes, *TP53* deletions, and mutations (11, 12). Mechanistic studies have highlighted oncogene enrichment (such as *MYC* and *EGFR*) in AML-specific eccDNAs. Storlazzi et al. demonstrated that MYC-containing DMs in leukemia cases are triggered by excision and amplification, which underpins the episome model (13). Abbate et al. confirmed MYC amplification in a large AML cohort via high-resolution genomic arrays, identifying associations with fusion transcripts and circular RNAs through DM-, homogeneously staining region (HSR)-, and ring chromosome-mediated mechanisms (14). Additionally, ATAC-seq-based indirect analysis revealed a positive correlation between eccDNA abundance and both normal hematopoietic differentiation and AML progression, suggesting their functional involvement in leukemogenesis (15). Here, we employ CIRCLE-seq and RNA sequencing (RNA-seq) to characterize eccDNA profiles in AML, providing direct evidence of their genomic and functional landscapes in this disease.

## Methods

### Patients and cell line

Bone marrow samples from all patients were collected from the Hematologic Biobank of the National Clinical Research Center for Hematologic Diseases, Jiangsu Biobank of Clinical Resources. Detailed information of the patients is presented in **Table 1**. Sample collection and research were conducted in accordance with the principles of the Declaration of Helsinki. This study was approved by the Academic Advisory Board of the First Affiliated Hospital of Soochow University.

**Table 1:** Characteristics of patients for CIRCLE-seq and RNA-seq.

ID	Sex	Age	FAB	Karyotype	Date of initial diagnosis	Date of sampling	Sample status	HB (g/L)	PLT (×10^9^/L)	WBC (×10^9^/L)	Blast in BM (%)	Mutations	Induction therapy	eccDNA counts
DAW	M	25	M3	46, XY, t(15;17) (q22;q12)[2]/46, idem, +r[inc2]/46, XY[6]	NA	9/1/2008	NA	NA	NA	NA	NA	NA	ATRA+ATO	15,522
YSH	M	52	M3	46, XY, t(15;17) (q24;q21)[6]/46, XY[4]	8/13/2019	8/13/2019	Pretreatment	88	35	9.09	88.4	ASXL1 p.H630fs	ATRA+ATO	17,939
CSQ	F	22	M3	NA	12/12/2019	12/14/2019	Pretreatment	99	39	107	83	NA	ATRA+ATO	63,274
LYY	M	32	M3	NA	10/10/2019	10/10/2019	Pretreatment	86	18	119.58	72	NA	ATRA+ATO	21,119
MJL	M	41	M1	46, XY[10]	1/11/2018	1/11/2018	Pretreatment	130	119	5.3	62.5	CEBPAdim, FLT3-ITD, NOTCH1 p.Q2391*, WT1 p.R462G, WT1 p.A211fs	IA	5,301
WGM	F	49	M2	46, XX[10]	1/17/2018	1/17/2018	Pretreatment	61	108	58.65	5.5	NPM1c, DIT3A p.K238*, IDH2 p.R140Q, IAK1 p.R343W, STAG2 p.I1102T	DAC+IA+G-CSF	8,341
TZX	F	33	M2	46, XX[10]	3/8/2018	3/8/2018	Pretreatment	91	119	0.9	52	IDH1 p.R132C, NPM1c, CSMD1 p.E1039D	IA	8,172
ZDM	F	42	NA	46, XX, add(1)(p36), del(5)(q13q34), −7, −8, +r, +M1[5]/46,X,-X, add(1), add(2)(q37),del(5),del(7)(q22q34),−8,+r,+M1[4]/47,XX,−1,add(2),del(5),del(7),−8,+r,+M1,+M2[1]	NA	5/15/2017	NA	NA	NA	NA	NA	NA	NA	1,847
CLP	M	61	NA	44, XY, −8, −9, −17, +M1, dmin1-2[8]/48, idem, +4, +14, +14, +19, −21, +M2[1]/46, XY[1]	5/21/2018	5/21/2018	Pretreatment	61	18	45.24	85.5	TP53 mutation	DAC+IA+G-CSF	45,539
ZYM	M	55	M1	46, XY, t(8,21)[10]	3/9/2018	3/9/2018	Pretreatment	78	108	37.58	81.5	−	DAC+IA+G-CSF	11,515
GY	F	43	M4E0	46, XY, inv(16)(p13q22)[10]	1/24/2018	1/24/2018	Pretreatment	70	24	44.3	37.5	NRAS p.Q61K	IA	3,486

NA, not available; −, negative; FAB, French-American-British classification of acute leukemias; HB, hemoglobin; PLT, platelet count; WBC, white blood cell count; ATRA, all-trans retinoic acid; ATO, arsenic trioxide; IA, idarubicin + cytarabine; DAC, decitabine; G-CSF, granulocyte colony-stimulating factor.

The HEL cell line was obtained from the ATCC and cultured in our laboratory with 1640 medium with 10% FBS and 1% penicillin/streptomycin and in a CO_2_ cell incubator.

### eccDNA identification from CIRCLE-seq

Total DNA was extracted from the cells using the high molecular weight (HMW) genomic DNA extraction kit (Magnetic Animal Tissue Genomic DNA Kit, Tiangen Beijing, China). A quantity of 1.5 μg of total DNA per sample was used in the subsequent steps. Elimination of linear chromosomal DNA was achieved by digestion with Plasmid-Safe ATP-Dependent DNase (California, USA, Biosearch, E0054) at 37°C for 24 h. Successful removal of linear DNA was confirmed by PCR using specific primers. Then, the eccDNA-enriched DNA was amplified with phi29 polymerase (Nanjing, China, Vazyme, N106) and random hexamer oligonucleotides. The DNA products were amplified by shearing phi29 (200–500 bp) with a focused ultrasound instrument (Bioruptor™, Diagenode Ghent, Belgium). The resulting eccDNA was purified with the MolPure^®^ Gel Extraction Kit (Shanghai, China, Yeasen, 19101) and subjected to library construction with the next-generation sequencing (VAHTS Universal DNA Library Prep Kit for Illumina V3, ND607). The samples were then cleaned up with DNA clean beads and analyzed on an Agilent 2100 Bioanalyzer (California, USA, Agilent) to measure fragment size distribution (approximately 150 bp). The qualified libraries were sequenced on Illumina NovaSeq 6000.

Fastq files were assessed for quality control using FastQC (v. 0.11.9). Clean reads were then aligned to reference genomes using BWA (v. 0.7.17). The resulting SAM files were sorted and processed by Samtools (v. 1.3.1) to generate the required input format for Circle-MAP. The eccDNA molecules were identified using Circle-MAP (v.1.1.4) with the following key parameters: –nhits 10 (number of realignment attempts), –cut_off 0.99 (probability cutoff for considering a soft-clipped read as realigned), –min_sc 5 (minimum soft-clipped length to attempt realignment), –gap_open 5 (gap open penalty in the position-specific scoring matrix), –gap_ext 1 (gap extension penalty), and –mapq 20 (minimum mapping quality allowed in the supplementary alignments). Genomic annotation of the identified eccDNAs was performed using Bedtools (v. 2.17.0). Differential eccDNA formation analysis was conducted using DESeq2. GO/KEGG and DisGeNET Disease enrichment were performed using the clusterProfiler and enrichR packages, respectively. Data with an absolute value of log2FoldChange greater than 1 and a *p*-value less than 0.05 were retained for subsequent analysis.

### RNA-seq

Total RNA was extracted from the cells using TRIzol reagent. mRNA was isolated and fragmented using mRNA Capture Beads (VAHTS mRNA Capture Beads, N401). The resultant RNA samples were reverse-transcribed for library construction using the NEB Next^®^ VAHTS Universal V6 RNA-seq Library Prep Kit for Illumina (NR604). The libraries were purified with DNA clean beads (VAHTS DNA Clean Beads, N411) for size selection of 200–300 bp fragments and analyzed on an Agilent 2100 Bioanalyzer (California, USA, Agilent) to measure fragment size distribution. The qualified libraries were sequenced on the BGI DNBSEQ-T7 platform.

Fastq files were assessed for quality control using FastQC (v. 0.11.9). The adapters were removed from the raw reads with Trim Galore (v. 1.18), and the cleaned reads were mapped to the human reference genome (hg38) with STAR (v. 2.7.10). SAM files were converted to BAM format using Samtools (v. 1.16.1). Gene expression levels and differential expression were calculated using HTseq-count (v. 2.0.2).

Differential expression analysis was performed using DESeq2, which applied an internal normalization procedure using the median-of-ratios method to account for differences in library size. Statistically significant results were defined as those with an absolute log2FoldChange greater than 1 and a *p*-value less than 0.05, and these were retained for all subsequent analyses.

### Survival analysis in TCGA-AML datasets

Differentially expressed eccDNAs (|log2FC| > 1, *p* < 0.05) from CIRCLE-seq data and differentially expressed genes (DEGs; |log2FC| > 1, *p* < 0.05) from RNA-seq data were obtained by comparing AML samples to healthy controls. The upregulated and downregulated genes carried by differential eccDNAs that overlapped with DEGs were further classified based on their annotations and literature evidence regarding their associations with tumors (especially hematological malignancies) to prioritize functionally relevant candidates.

Prognostic analysis of candidates was performed using RNA-seq data from the TCGA-LAML cohort (*n* = 173) accessed via the GEPIA2 web server (http://gepia2.cancer-pku.cn/). Patients were stratified into high- and low-expression groups based on the median expression level of each gene, and overall survival (OS) was compared using a log-rank test. Only those with statistically significant prognostic values (*p* < 0.05) were included in the Kaplan–Meier survival analysis.

### Statistical analysis

All statistical tests were implemented in R-4.2.2. The difference comparison of two groups was performed by the Wilcoxon test. A Pearson’s correlation test was used for correlation analysis. A *p*-value < 0.05 indicated statistical significance.

## Results

**Figure 1:**
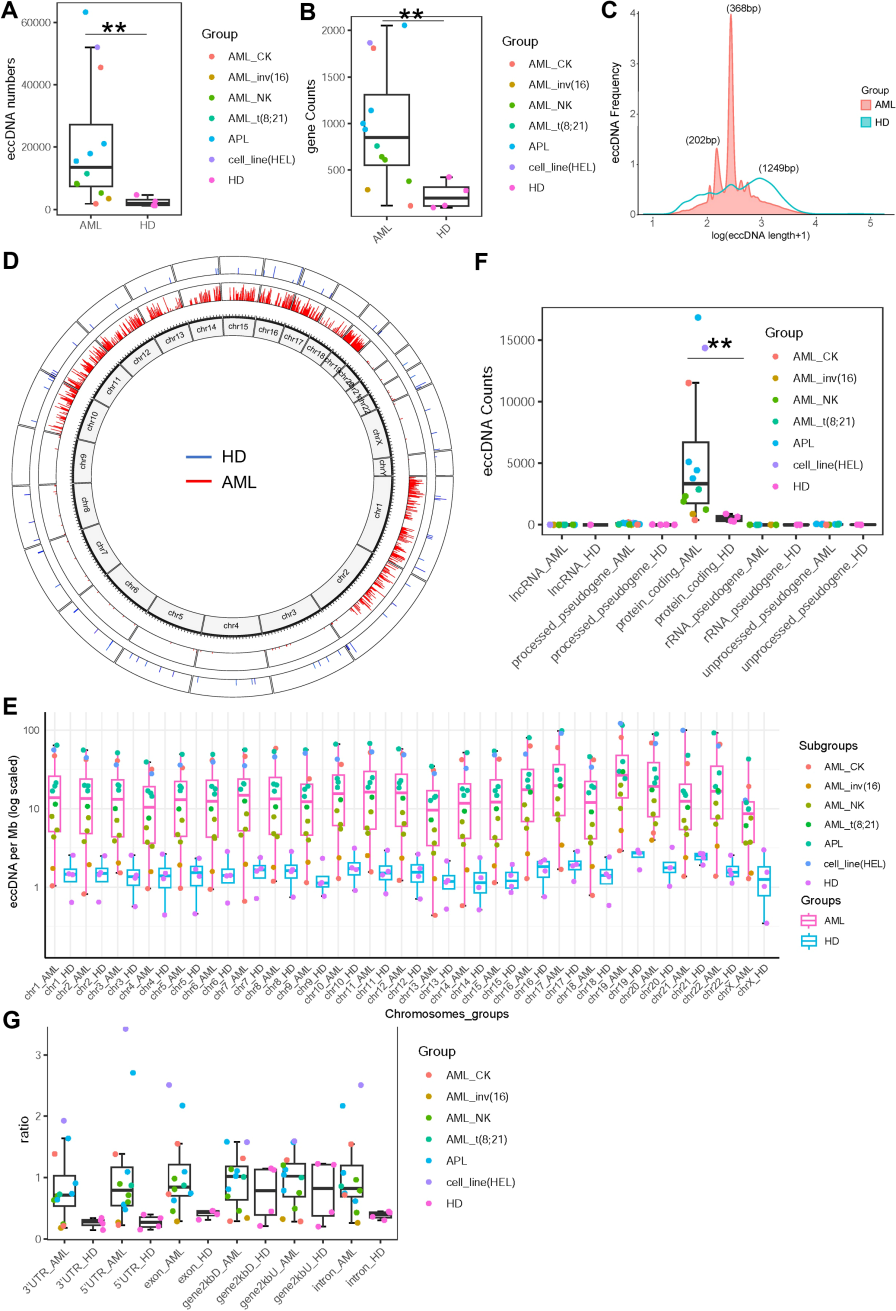
Characteristics of extrachromosomal circular DNAs (eccDNAs) in acute myeloid leukemia (AML) and healthy controls. **(A)** Comparison of eccDNA counts between AML patients (*n* = 12) and healthy individuals (*n* = 4). Median values (13,518 *vs*. 1,964, *p* = 0.00977) are shown. **(B)** Comparison of the number of genes involved in eccDNAs between the two groups. Median values (848 *vs*. 201 genes, *p* = 0.0026) are indicated. **(C)** Size distribution of eccDNAs. AML patients exhibited five distinct peaks, with ~368 and ~202 bp being the most frequent, while healthy controls showed a relatively uniform distribution. **(D)** Chromosomal localization of eccDNAs. AML-derived eccDNAs (red) showed non-random distribution, primarily enriched on chromosomes 1, 2, 10–19, whereas healthy controls exhibited relatively random distribution (blue). **(E)** eccDNA density (per Mb) normalized by chromosomal length, showing significantly higher density in all chromosomes of AML compared to healthy controls. **(F)** Functional annotation of genes harbored by eccDNAs, indicating both groups predominantly contained protein-coding genes with a significantly greater number in AML samples. **(G)** Regional distribution of eccDNAs relative to gene structures. AML-derived eccDNAs were enriched within 2 kb upstream/downstream of transcription start sites (gene2kbU/D), 5′UTR, 3′UTR, exon, and intron regions, while control eccDNAs showed lower abundance in these regions.

To investigate eccDNA in AML, we performed CIRCLE-seq on bone marrow cells from 4 healthy individuals and 12 AML patients (including 4 APL cases, 3 with normal karyotype [AML_NK], 2 with complex karyotype [AML_CK], 1 with t (8,21), 1 with inv (16), and 1 with cell line HEL). A methodological workflow is presented in **Supplementary Figure S1**. Patient characteristics are detailed in **Table 1**. Compared to healthy controls, AML patients exhibited a significant increase in eccDNA counts (median: 13,518 *vs*. 1,964, *p* = 0.00977; **Figure 1A**) and gene involvement (median: 848 *vs*. 201 genes, *p* = 0.0026; **Figure 1B**). Size distribution analysis of the eccDNAs revealed distinct patterns: AML samples showed five prominent size peaks at 149, 202, 368, 568, and 742 bp (with 202 and 368 bp being the most frequent sizes), whereas healthy controls had a more uniform distribution with flat peaks (**Figure 1C**), suggesting subtype-specific biogenesis pathways in AML.

Subsequently, chromosomal mapping demonstrated non-random localization of AML-derived eccDNA, with significant enrichment on chromosomes 1, 2, and 10–19, in contrast to the relatively random distribution in healthy controls (**Figure 1D**). When normalized by chromosomal length, eccDNA density (per megabase pairs) was significantly higher in all chromosomes of AML compared to healthy controls (**Figure 1E**). Functional annotation showed that while both groups enriched protein-coding genes, AML samples had a greater number of involved genes (**Figure 1F**). Notably, AML-derived eccDNA was preferentially located within 2 kb upstream/downstream of transcription start site regions (gene2kbU/D) as well as gene regulatory regions (5′UTR, 3′UTR, exons, introns), contrasting with lower enrichment in the control samples (**Figure 1G**), supporting a role in transcriptional modulation.

**Figure 2:**
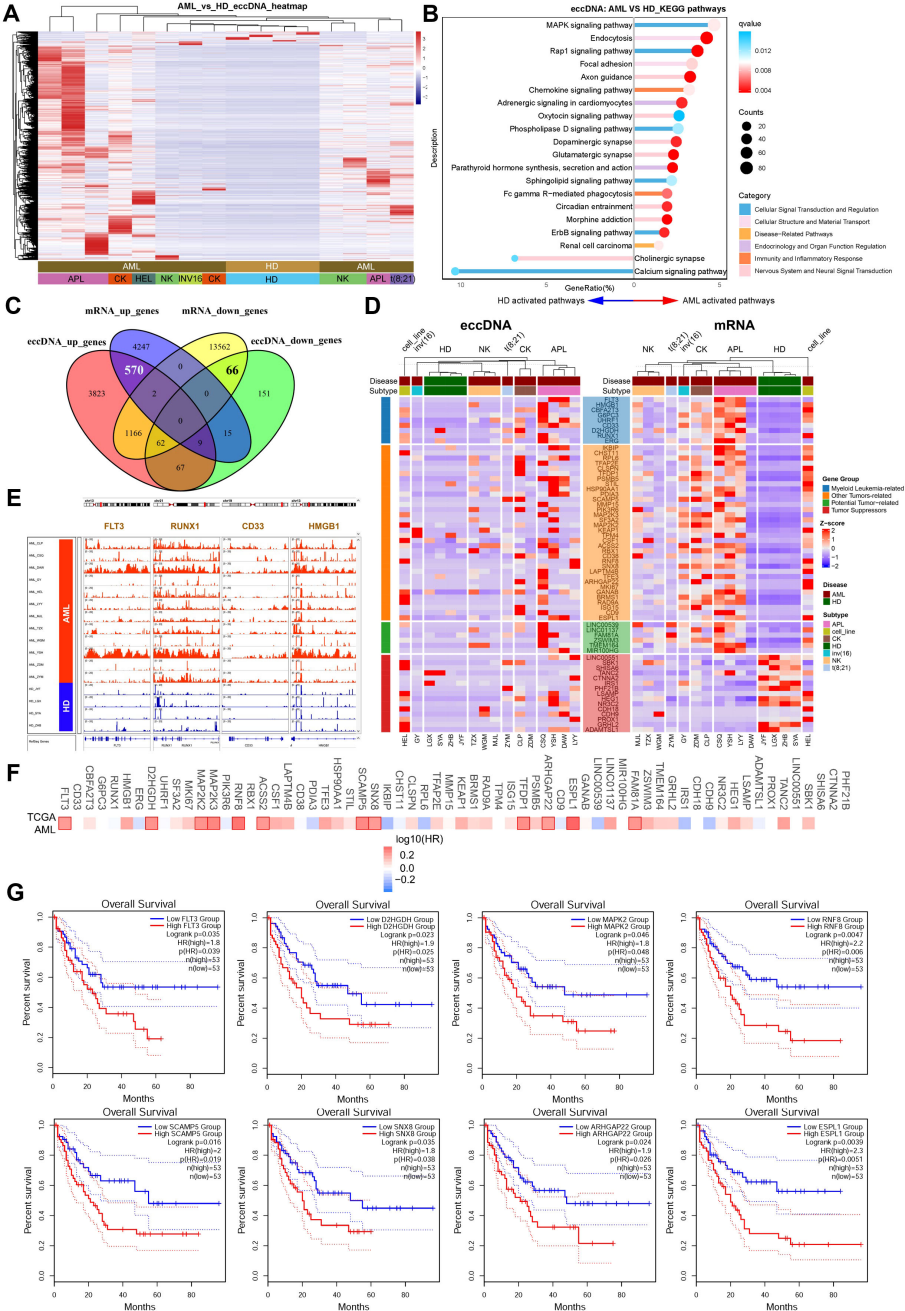
Functional enrichment and integrative analysis of differentially expressed genes using eccDNA-seq and RNA-seq data. **(A)** Heatmap displaying distinct eccDNA-associated gene profiles between AML patients and healthy controls. **(B)** KEGG pathway enrichment analysis. Significantly activated pathways in AML included MAPK, Rap1, phospholipase D, sphingolipid, ErbB signaling, neurological pathways, and immune-inflammatory pathways, while only cholinergic synapse and calcium signaling pathways were enriched in controls. **(C)** The Venn plot shows an integrative analysis of eccDNA sequencing and RNA-seq data, which identified 570 upregulated and 66 downregulated genes at both the eccDNA and mRNA levels. **(D)** Functional annotation of differentially expressed genes. Upregulated genes included myeloid leukemia-associated genes, tumor progression-related genes, and potential oncogenes; downregulated genes included candidate tumor suppressors. **(E)** IGV visualization showing significant enrichment of eccDNA at genomic loci of tumor-related genes (e.g., *FLT3, RUNX1, CD33, HMGB1*). **(F, G)** Prognostic analysis using TCGA datasets (GEPIA2) revealed that high expression of tumor-related genes (*FLT3, D2HGDH, MAPK2*, etc.) correlated with poor outcomes in AML patients (median cutoff).

Next, we performed functional enrichment and integrative analysis of the differentially enriched genes carried by eccDNAs between the AML and healthy controls. Heatmap analysis revealed striking differences in eccDNA-associated gene profiles between AML patients and controls (**Figure 2A**). KEGG pathway enrichment identified significantly activated signaling networks in AML, including MAPK, Rap1, phospholipase D, sphingolipid, and ErbB signaling pathways, some neurological pathways (axon guidance, oxytocin/dopaminergic/glutamatergic synapses, circadian rhythm regulation, morphine addiction), and some immune-inflammatory pathways (chemokine signaling and Fcγ receptor-mediated phagocytosis). In contrast, only cholinergic synapse and calcium signaling pathways were enriched in healthy controls (**Figure 2B**). In addition, Gene Ontology (GO) enrichment analysis with upregulated eccDNA-carrying genes in AML revealed enrichment in specific biological processes (BPs), including “cell growth,” “Ras protein signal transduction,” “histone modification,” and “ERBB signaling pathway”; cellular components (CCs), including “histone deacetylase complex,” “SWI/SNF superfamily-type complex,” and “protein phosphatase type 2A complex”; and molecular function (MFs), including “GTPase regulator activity,” “protein serine kinase activity,” and “14-3–3 protein binding” (**Supplementary Figure S2**). In contrast, GO analysis of downregulated eccDNA-carrying genes identified enriched BPs, specifically “regulation of gamma-delta T-cell activation,” “gamma-delta T-cell differentiation,” “T-cell selection,” and “T-cell lineage commitment” (**Supplementary Figure S3**). Furthermore, DisGeNET Disease enrichment analysis showed that most terms related to blood cell counts were upregulated, including those for platelets, granulocytes, neutrophils, eosinophils, and basophils (**Supplementary Figure S4**). In contrast, downregulated disease terms were primarily associated with non-hematological conditions, such as “post-traumatic stress disorder,” “intelligence,” and “neurodevelopmental disorders” (**Supplementary Figure S5**). The detailed results of GO, KEGG, and DisGeNET Disease enrichment analyses have been deposited in **Supplementary Table S1.**

To determine the consequences of DNA circularization on gene expression, we performed total RNA-seq on our AML and healthy cases. Integrative analysis of CIRCLE-seq and RNA-seq data has identified 570 genes upregulated and 66 genes downregulated at both the eccDNA and mRNA levels (**Figure 2C**). Functional annotation of upregulated genes highlighted at least 9 myeloid leukemia-associated genes (*FLT3, RUNX1, CD33, HMGB1, ERG, CBFA2T3, G6PC3, D2HGDH, UHRF1*), 35 other tumor progression-related genes (e.g., *MAP2K2/3, MKI67, KEAP1, HSP90AA1*), and 6 potential oncogenes (e.g., *LINC00539, LINC01137*). In contrast, downregulated genes included at least 15 candidate tumor suppressors (e.g., *CDH18, HEG1*) (**Figure 2D**). In addition, IGV visualization confirmed significant enrichment of eccDNA at genomic loci of tumor-related genes such as *FLT3, RUNX1, CD33*, and *HMGB1* (**Figure 2E**). Finally, prognostic analysis using TCGA datasets (https.//gepia2.cancer-pku.cn) showed that high expression (median cutoff) of these tumor-related genes including *FLT3, D2HGDH, MAPK2, RNF8, SCAMP5, SNX8, ARHGAP22*, and *ESPL1* correlated with poor outcomes in AML patients (**Figures 2F, G**).

## Discussion

Extrachromosomal circular DNA has emerged as a pivotal mediator of genomic plasticity in cancer, yet its biological significance in hematological malignancies, particularly acute myeloid leukemia, remains vastly under-characterized compared to solid tumors. To our knowledge, this study has been the first to directly profile eccDNA in AML using CIRCLE-seq, complemented by RNA-seq, thereby providing high-resolution insights into its genomic features, functional impact, and clinical relevance.

In solid tumors, eccDNA is well-established as a driver of oncogene amplification, therapeutic resistance, and tumor heterogeneity. For instance, Kim et al. (2) demonstrated that eccDNA (especially ecDNA, megabase-scale subtypes) was prevalent across cancers such as glioblastoma and colorectal cancer, often harboring oncogenes (e.g., *MYC* and *EGFR*) to drive aggressive phenotypes. Similarly, Koche et al. (8) showed that eccDNA mediates oncogenic genome remodeling in neuroblastoma by amplifying lineage-specific drivers. Our findings aligned with these studies in three key ways: first, AML cells exhibited significantly higher eccDNA burden and gene involvement than healthy controls (median 13,518 *vs*. 1,964 eccDNAs; 848 *vs*. 201 genes), reflecting a general trend of eccDNA accumulation in malignant cells and presenting a potential link between eccDNA biogenesis and the genomic instability inherent in leukemia (such as complex karyotype). Second, the non-random chromosomal distribution of eccDNA in AML cells, with enrichment on chromosomes 1, 2, and 10–19, and the striking localization near transcription start sites and regulatory regions strongly suggested a selective mechanism favoring the circularization of genomic segments with high regulatory potential. This finding aligned with the emerging concept of eccDNAs functioning as mobile transcriptional enhancers or “super-enhancers” that can decouple oncogene regulation from their native chromosomal context, a mechanism observed in solid tumors (9). Third, integrative analysis identified 570 genes upregulated at both the eccDNA and mRNA levels, including myeloid leukemia drivers (*FLT3, RUNX1, CD33*) and oncogenes (*MAP2K2/3, LINC00539*), mirroring the “eccDNA–oncogene amplification” axis observed in solid tumors.

Notably, however, critical discrepancies emerged when comparing AML to solid tumors and even other hematological neoplasms. For example, DMs (a large eccDNA subtype) are rare in myeloid neoplasms (11, 12), whereas they are common in solid tumors like lung cancer. Our study did not detect prominent DM-like signals; instead, AML eccDNAs showed distinct size peaks at 202 and 368 bp—consistent with microDNA (100–300 bp) and small eccDNA subtypes, which were thought to arise from DNA repair rather than chromosomal fragmentation (4, 6). This discrepancy might stem from two factors: 1) there were biological differences in genomic instability mechanisms between solid tumors and AML, and 2) technical resolution—CIRCLE-seq, unlike whole-genome sequencing (WGS), was optimized for detecting small eccDNAs, whereas prior studies of myeloid DMs relied on cytogenetics or low-resolution arrays (13), which might have missed small subtypes. Another key discrepancy with prior studies was the non-random chromosomal localization of AML eccDNAs. This difference might arise from at least the following three points: 1) non-random distribution of cancer-related genes and fragile sites, which might be prone to DNA breakage and circularization; 2) differences in epigenetic modifications: active transcriptional regions and open chromatin states might facilitate eccDNA formation; and 3) genomic instability: “high fragility tendency” of AML chromosomes.

In AML, prior work on eccDNA has been indirect and limited. Zeng et al. showed that eccDNA abundance correlated with normal hematopoietic differentiation and AML progression, suggesting a functional role in lineage commitment through the analysis of ATAC-seq. Our direct sequencing data validated and extended this observation: healthy controls exhibited low eccDNA counts with uniform size distribution and enrichment in pathways critical for normal hematopoiesis [e.g., calcium signaling, which regulates hematopoietic stem cell (HSC) self-renewal (16)]. Additionally, another notable similarity was the correlation between eccDNA and poor prognostic markers: both studies linked eccDNA enrichment to genes associated with adverse AML outcomes (e.g., *FLT3, D2HGDH*).

The functional enrichment of our eccDNA data provided compelling evidence for its role in leukemogenesis. The significant activation of oncogenic pathways such as MAPK, Rap1, and ErbB signaling in AML-associated eccDNAs mirrored findings in solid tumors, where these pathways were frequently co-opted for proliferation and survival (17). This convergence highlighted a fundamental similarity in eccDNA-driven oncogenesis across cancer types. However, we also observed unique enrichments, including several neurological pathways. This observation might not be incidental but could reflect shared signaling networks between hematopoietic and neural development, or the involvement of these pathways in the self-renewal properties of leukemia stem cells. Additionally, GO enrichment in “histone modification” and CCs like “histone deacetylase complex” and “SWI/SNF superfamily-type complex” implies that eccDNA might modulate chromatin accessibility via epigenetic and chromatin-remodeling machineries—an emerging mechanism by which eccDNA regulates gene expression in cancer. The enrichment of MFs including “GTPase regulator activity” and “protein serine kinase activity” further confirmed eccDNA’s role in activating dysregulated intracellular signaling networks that sustain AML malignancy. In contrast, GO analysis identified downregulated biological processes including gamma-delta T-cell activation and differentiation and T-cell selection and lineage commitment, which directly linked eccDNA to the immune dysregulation of AML cells. γδ T cells were critical innate immune effectors against leukemic cells, and the downregulation of their functional pathways suggested that eccDNA might indirectly suppress anti-leukemic immunity. This finding provided a molecular clue to how AML cells evaded immune surveillance, potentially by depleting eccDNA-carrying genes that support γδ T-cell-mediated immunity. DisGeNET enrichment results further contextualize eccDNA’s impact on AML pathophysiology: upregulated terms related to blood cell counts (e.g., platelets, granulocytes, neutrophils) aligned with the typical hematological abnormalities observed in AML patients (e.g., peripheral blood cytopenias or leukocytosis), reflecting eccDNA’s specific regulatory role in the hematopoietic system. Meanwhile, downregulated terms associated with “post-traumatic stress disorder,” “intelligence,” and “neurodevelopmental disorders” were likely indirect consequences of AML-induced hematopoietic dysfunction rather than direct targets of eccDNA. This reinforced that eccDNA primarily exerted its functional effects on the malignant hematopoietic phenotype rather than non-hematological processes. Collectively, these enrichment results functionally validated that eccDNA contributed to AML pathogenesis by “activating oncogenic pathways and inhibiting anti-leukemic immunity” and provided clear directions for subsequent investigations into the specific regulatory mechanisms of eccDNA in AML.

The integration of CIRCLE-seq with RNA-seq was pivotal, allowing us to move beyond correlation to identify 570 genes concordantly upregulated at both the DNA circularization and transcriptional levels. This gene set was heavily enriched with myeloid leukemia drivers (e.g., *FLT3, RUNX1, CD33*) and other tumor-promoting genes, providing a direct mechanistic link between eccDNA presence and the transcriptional dysregulation that fuels AML. The poor prognosis associated with high expression of these genes in independent TCGA cohorts further solidified their clinical relevance.When contextualized within the broader field, our findings in AML both aligned with and diverged from observations in normal hematopoiesis. Studies in normal hematopoietic cells have reported the presence of eccDNA, with its abundance potentially correlating with differentiation states (10). This suggested a potential physiological role for eccDNA in normal cellular processes. The stark contrast lay in functional output: in normal hematopoiesis, eccDNA might contribute to regulated gene expression for differentiation, whereas in AML, it was hijacked to amplify and deregulate oncogenes, driving malignant transformation. This divergence might stem from distinct selective pressures—the pervasive genomic instability and strong clonal selection in AML favored the retention and expansion of eccDNA that provided a proliferative advantage.

However, our study had several limitations that must be acknowledged. The primary constraint was the small cohort size, which precluded robust stratification across the diverse molecular and cytogenetic subtypes of AML and limited the generalizability of our findings. Furthermore, our methodology was subject to the technical biases inherent to CIRCLE-seq, including potential amplification biases from phi29 polymerase and the challenges in mapping circular DNA—both of which might influence the quantitative and qualitative aspects of our eccDNA landscape. Finally, our study was observational: while the integrative analysis strongly suggests functionality, it does not prove causality.These limitations charted a clear course for future research. Validation in larger, independent AML cohorts was essential to confirm our findings and explore associations with specific genetic subtypes and clinical outcomes. Functional assays were the crucial next step; CRISPR-based technologies to selectively deplete or amplify specific eccDNAs, followed by phenotypic assays, would be necessary to establish their direct role in leukemogenesis. Investigating the dynamics of eccDNA during disease progression and in response to therapy could unveil its role in therapeutic resistance. Finally, technological advancements, such as long-read sequencing of eccDNA, would provide deeper insights into its full structural complexity.

In conclusion, this study provided the first direct characterization of the eccDNA landscape in AML, linking its accumulation to oncogenic pathway activation, transcriptional dysregulation, and poor clinical outcomes. While our findings aligned with broader themes of eccDNA in cancer (e.g., oncogene amplification, genomic plasticity), they also revealed AML-specific features (e.g., non-random chromosomal localization). Addressing the study’s limitations through larger cohorts, improved sequencing technologies, and functional assays would solidify eccDNA’s role as a diagnostic biomarker and therapeutic target in AML. Ultimately, unraveling the eccDNA regulatory network might open new avenues for precision medicine in this aggressive hematological malignancy.

## Data Availability

The raw data of CIRCLE-seq and RNA-seq are available in the CNCB database: https://ngdc.cncb.ac.cn, accession number HRA013780.

## References

[1] Yan Y Guo G Huang J Gao M Zhu Q Zeng S . Current understanding of extrachromosomal circular DNA in cancer pathogenesis and therapeutic resistance. J Hematol Oncol. (2020) 13(1):124. doi: 10.1186/s13045-020-00960-9, PMID: 32928268 PMC7491193

[2] Kim H Nguyen NP Turner K Wu S Gujar AD Luebeck J . Extrachromosomal DNA is associated with oncogene amplification and poor outcome across multiple cancers. Nat Genet. (2020) 52:891. doi: 10.1038/s41588-020-0678-2, PMID: 32807987 PMC7484012

[3] Yang L Jia R Ge T Ge S Zhuang A Chai P . Extrachromosomal circular DNA: biogenesis, structure, functions and diseases. Signal Transduct Tar. (2022) 7(1):342. doi: 10.1038/s41392-022-01176-8, PMID: 36184613 PMC9527254

[4] Paulsen T Kumar P Koseoglu MM Dutta A . Discoveries of extrachromosomal circles of DNA in normal and tumor cells. Trends Genet. (2018) 34:270–8. doi: 10.1016/j.tig.2017.12.010, PMID: 29329720 PMC5881399

[5] Paulsen T Shibata Y Kumar P Dillon L Dutta A . Small extrachromosomal circular DNAs, microDNA, produce short regulatory RNAs that suppress gene expression independent of canonical promoters. Nucleic Acids Res. (2019) 47:4586–96. doi: 10.1093/nar/gkz155, PMID: 30828735 PMC6511871

[6] Shibata Y Kumar P Layer R Willcox S Gagan JR Griffith JD . Extrachromosomal microDNAs and chromosomal microdeletions in normal tissues. Science. (2012) 336:82–6. doi: 10.1126/science.1213307, PMID: 22403181 PMC3703515

[7] Kloosterman WP Guryev V van Roosmalen M Duran KJ de Bruijn E Bakker SC . Chromothripsis as a mechanism driving complex structural rearrangements in the germline. Hum Mol Genet. (2011) 20:1916–24. doi: 10.1093/hmg/ddr073, PMID: 21349919

[8] Koche RP Rodriguez-Fos E Helmsauer K Burkert M MacArthur IC Maag J . Extrachromosomal circular DNA drives oncogenic genome remodeling in neuroblastoma. Nat Genet. (2020) 52:29. doi: 10.1038/s41588-019-0547-z, PMID: 31844324 PMC7008131

[9] Wu S Turner KM Nguyen N Raviram R Erb M Santini J . Circular ecDNA promotes accessible chromatin and high oncogene expression. Nature. (2019) 575:699. doi: 10.1038/s41586-019-1763-5, PMID: 31748743 PMC7094777

[10] Møller HD Mohiyuddin M Prada-Luengo I Sailani MR Halling JF Plomgaard P . Circular DNA elements of chromosomal origin are common in healthy human somatic tissue. Nat Commun. (2018) 9(1):1069. doi: 10.1038/s41467-018-03369-8, PMID: 29540679 PMC5852086

[11] Huh YO Tang G Talwalkar SS Khoury JD Ohanian M Bueso-Ramos CE . Double minute chromosomes in acute myeloid leukemia, myelodysplastic syndromes, and chronic myelomonocytic leukemia are associated with micronuclei, or amplification, and complex karyotype. Cancer Genet-Ny. (2016) 209:313–20. doi: 10.1016/j.cancergen.2016.05.072, PMID: 27318442

[12] Koduru P Chen WN Haley B Ho K Oliver D Wilson K . Cytogenomic characterization of double minute heterogeneity in therapy related acute myeloid leukemia. Cancer Genet-Ny. (2019) 238:69–75. doi: 10.1016/j.cancergen.2019.08.001, PMID: 31425928

[13] Storlazzi CT Lonoce A Guastadisegni MC Trombetta D D'Addabbo P Daniele G . Gene amplification as double minutes or homogeneously staining regions in solid tumors: Origin and structure. Genome Res. (2010) 20:1198–206. doi: 10.1101/gr.106252.110, PMID: 20631050 PMC2928498

[14] L'Abbate A Tolomeo D Cifola I Severgnini M Turchiano A Augello B . MYC-containing amplicons in acute myeloid leukemia: genomic structures, evolution, and transcriptional consequences. Leukemia. (2018) 32:2304–4. doi: 10.1038/s41375-018-0177-y, PMID: 29467491 PMC6170393

[15] Zeng T Huang W Cui L Zhu P Lin Q Zhang W . The landscape of extrachromosomal circular DNA (eccDNA) in the normal hematopoiesis and leukemia evolution. Cell Death Discov. (2022) 8. doi: 10.1038/s41420-022-01189-w, PMID: 36171187 PMC9519993

[16] Ho KYL An K Carr RL Dvoskin AD Ou AYJ Vogl W . Maintenance of hematopoietic stem cell niche homeostasis requires gap junction-mediated calcium signaling. Proc Natl Acad Sci U S A. (2023) 120:e2303018120. doi: 10.1073/pnas.2303018120, PMID: 37903259 PMC10636368

[17] Verhaak RGW Bafna V Mischel PS . Extrachromosomal oncogene amplification in tumour pathogenesis and evolution. Nat Rev Cancer. (2019) 19:283–8. doi: 10.1038/s41568-019-0128-6, PMID: 30872802 PMC7168519

