# Supplementary material for: Integrated CIRCLE-seq with RNA-seq to decipher the quantity, localization, and functional features of eccDNA in AML
Source: Front Oncol. 2025 Nov 7;15:1701989. doi: 10.3389/fonc.2025.1701989 (PMC12634341; doi:10.3389/fonc.2025.1701989)
Supplement: Supplementary file 1 [file DataSheet1.docx]

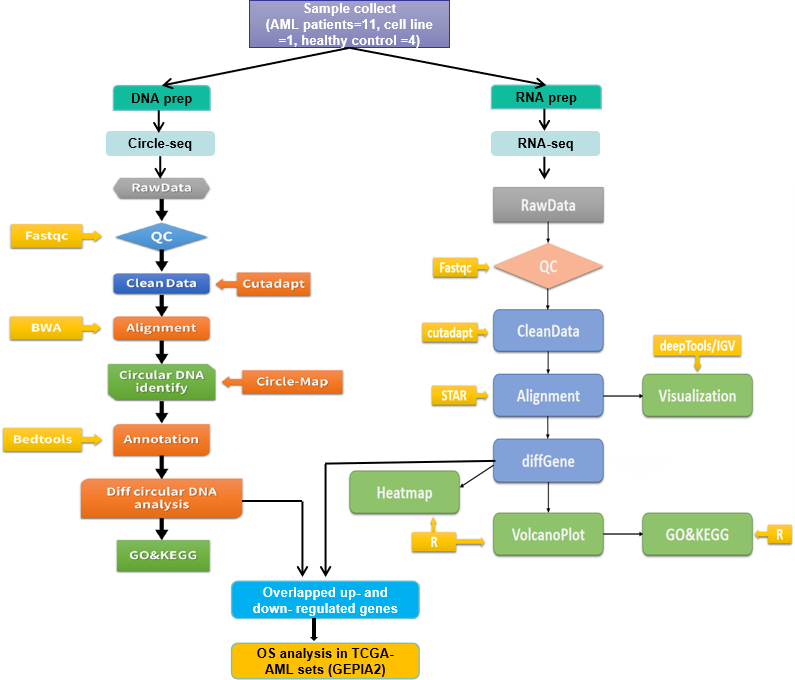


Supplementary Figure S1. A methodological workflow of integrate circle-seq and RNA-seq to decipher the landscape features of eccDNA in AML.


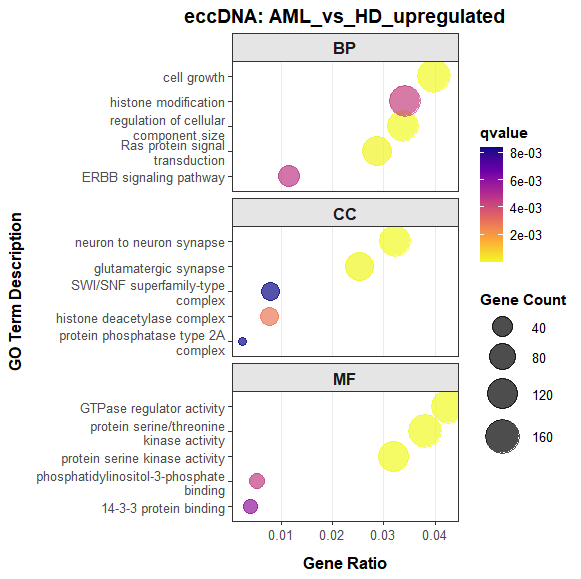


Figure S2. GO enrichment analysis of differential up-regulated eccDNA-derived genes in AML compared to the healthy donor (HD). Bubble plot showing the significant enriched Gene Ontology (GO) terms categorized by Biological Process (BP), Cellular Component (CC), and Molecular Function (MF).


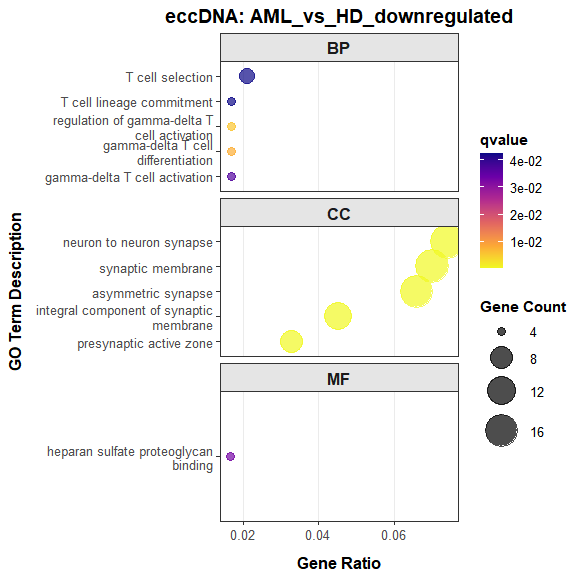


Figure S3. GO enrichment analysis of differential down-regulated eccDNA-derived genes in AML compared to the healthy donor (HD). Bubble plot showing the significant enriched Gene Ontology (GO) terms categorized by Biological Process (BP), Cellular Component (CC), and Molecular Function (MF).


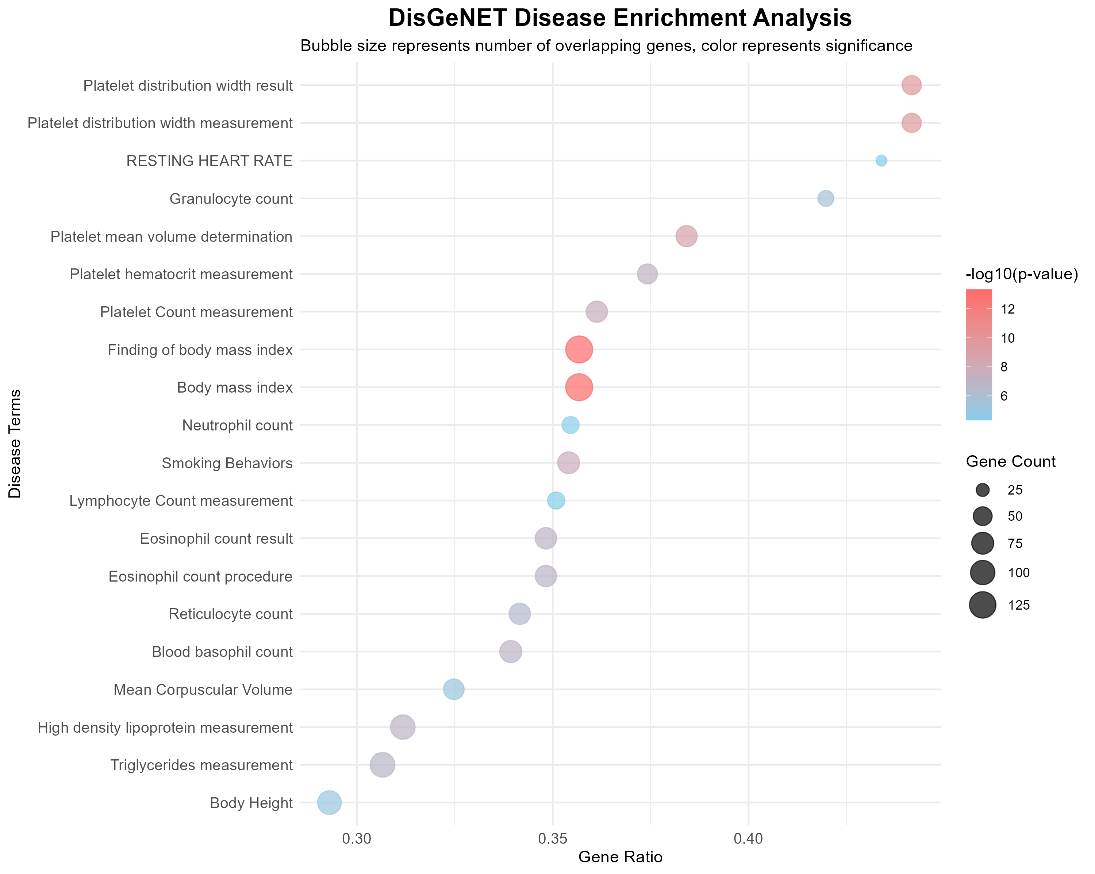


Figure S4. DisGeNET-based disease enrichment analysis of differential up-regulated eccDNA-derived genes in AML compared to the healthy donor (HD). Bubble plot showing the top disease terms with the most significant enrichment.


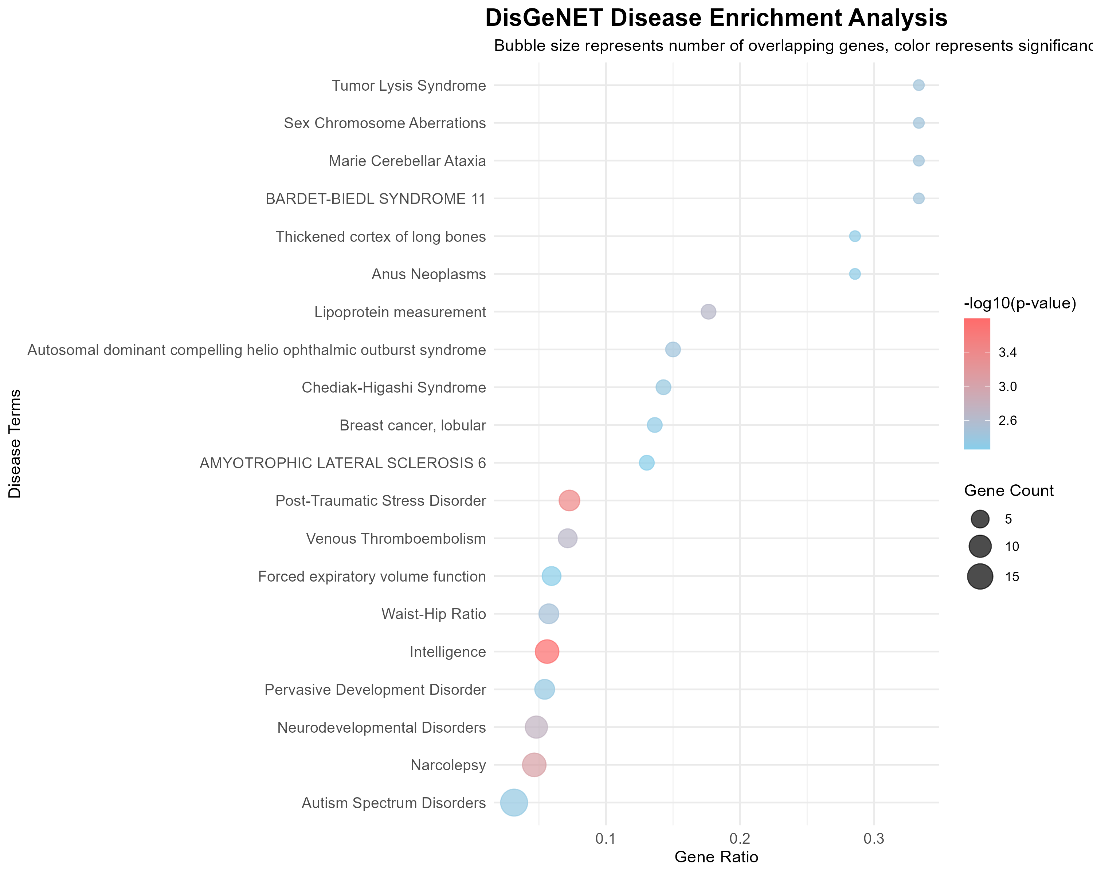


Figure S5. DisGeNET-based disease enrichment analysis of differential down-regulated eccDNA-derived genes in AML compared to the healthy donor (HD). Bubble plot showing the top disease terms with the most significant enrichment.
